# Differences in the clinical presentation, management, and in-hospital outcomes of acute aortic dissection in patients with and without end-stage renal disease

**DOI:** 10.1186/s12882-021-02432-9

**Published:** 2021-07-08

**Authors:** Jiahe Xie, Shan Zeng, Long Xie, Rongming Ding, Jing Hu, Hong Zeng, Weiling Lu, Yuhua Hu, Qingrui Li, Gaojun Zhong, Shiju Zhou, Ziyou Liu, Yulin Liao, Yiming Zhong, Dongming Xie

**Affiliations:** 1 1Department of Cardiology, First Affiliated Hospital, Key Laboratory of Prevention and Treatment of Cardiovascular and Cerebrovascular Diseases, Ministry of Education, Jiangxi Branch Center of National Geriatric Disease Clinical Medical Research Center, Gannan Medical University, Ganzhou, 341000 China; 2 Department of Geriatric, the Affiliated Ganzhou Hospital of Nanchang University, Ganzhou, 341000 China; 3 Department of Cardiology, the Affiliated Ganzhou Hospital of Nanchang University, Ganzhou, 341000 China; 4 Department of Cardiovascular, Jiangxi Provincial People’s Hospital Affiliated to Nanchang University, Nanchang, Jiangxi 330006 China; 5 Department of Cardiology, State Key Laboratory of Organ Failure Research, Nanfang Hospital, Southern Medical University, Guangzhou, 510515 China

**Keywords:** End-stage renal disease, Acute aortic dissection, Management, Mortality

## Abstract

**Background:**

Few studies have evaluated the clinical presentation, management, and outcomes of patients with end-stage renal disease (ESRD) presenting with acute aortic dissection (AAD) in real-world clinical practice. Thus, this study investigated the clinical characteristics, management, and outcomes of AAD patients with ESRD.

**Methods:**

A total of 217 patients were included. We evaluated the differences in the clinical features, management, and in-hospital outcomes of patients with and without a history of ESRD presenting with AAD.

**Results:**

A history of ESRD was present in 71 of 217 patients. Patients with ESRD had atypical clinical manifestations (*p* < 0.001) and were more likely to be managed medically compared with patients without ESRD (*p* = 0.002). Hypertension and type B aortic dissection were significantly more common among patients with ESRD. Moreover, patients with ESRD had lower leucocyte and platelet counts than patients without ESRD in laboratory findings (*p* < 0.001). However, hospitalization days and in-hospital mortality were similar between the two groups (*p* > 0.05). Multivariate analysis identified Type A aortic dissection as an independent predictor of in-hospital mortality among patients without ESRD (OR, 13.68; 95% CI, 1.92 to 98.90; *P* = 0.006).

**Conclusions:**

This study highlights differences in the clinical characteristics, management, and outcomes of AAD patients with ESRD. These patients usually have atypical symptoms and more comorbid conditions and are managed more conservatively. However, these patients have no in-hospital survival disadvantage over those without ESRD. Further studies are needed to better understand and optimize care for patients with ESRD presenting with AAD.

## Background

In clinical practice, acute aortic dissection (AAD) is a critical disease that must be urgently treated because of the associated high risk of developing life-threatening complications. Currently, the mortality of AAD ranges from 25 to 30% [1]. Type A aortic dissection constitutes a surgical emergency, and the mortality rates increase 1–2% per hour after symptom onset without surgical intervention [2]. For patients with type B aortic dissection, medical management is the most selected treatment, whereas surgical treatment or endovascular stenting are performed for patients with complications such as rupture, aortic expansion, and malperfusion syndrome [3–5].

Patients with end-stage renal disease (ESRD) are known to have a shorter life expectancy and a higher incidence of cardiovascular events than the average population. In addition, patients with ESRD combined with AAD have high mortality rates after both open surgical and endovascular thoracic aortic interventions [6]. However, few studies have evaluated the clinical presentation, management, and outcomes of AAD patients with ESRD in real-world clinical practice, especially in China. Thus, the purpose of this study was to explore the differences in clinical features, management, and in-hospital outcomes of AAD patients with and without a history of ESRD. Furthermore, we sought to determine patient characteristics associated with an increased risk of in-hospital mortality in a cohort with ESRD.

## Methods

### Study population and data explanation

Data on patients who were admitted to hospitals with a diagnosis of AAD between 1 May 2011–30 November 2018 were obtained from the clinical research database of the First Affiliated Hospital of Gannan Medical University, Jiangxi Provincial People’s Hospital Affiliated to Nanchang University and the Affiliated Ganzhou Hospital of Nanchang University. The identification of patients hospitalized for aortic dissection was first based on the International Classification of Diseases (ICD)-10 diagnosis code I71.0. We excluded patients who were not admitted for emergency treatment. In addition, patients with chronic dissection or dissections at unspecified sites were also excluded. Baseline characteristics, clinical presentation, treatment modality, in-hospital mortality, length of hospitalization, and some specific blood biochemical indexes were analyzed. Finally, patients with AAD were categorized into 2 groups: those with and those without a history of ESRD. In this study, only patients with normal renal function (no history of CKD) or early (stage 1 or 2) CKD) were included in the group without ESRD. According to 2012 KDIGO Clinical Practice Guideline for the Evaluation and Management of CKD, patients included in the ESRD group must have a history of chronic kidney disease for at least 3 months and meet the following two criteria: the presence of estimated GFR < 15 mL/min/1.73m^2^ and the need for renal replacement therapy (RRT: dialysis and/or transplant). Intermediate (stages 3a to 4) CKD represents different pathophysiological processes of CKD compared with early CKD or ESRD. Thus patients with intermediate (stages 3a to 4) CKD were not included in this study. GFR was estimated by the CKD-EPI formula.

### Statistical analyses

Statistical analysis was conducted using IBM SPSS Version 18.0 (SPSS Science, Chicago, IL, USA). Data are expressed as the means ± standard deviations (SDs) for continuous variables and were analyzed by Student’s t-test or the Mann–Whitney U test, while categorical variables are presented as n (%) and analyzed using the chi-square test or Fisher’s exact test. A two-sided *p*-value < 0.05 was considered statistically significant. Multiple logistic regression analysis was used to define the independent predictors associated with in-hospital mortality in AAD patients.

## Results

### Patient characteristics, clinical presentation, comorbidities, and laboratory and imaging findings in AAD patients with and without ESRD

A total of 217 patients admitted with a primary diagnosis of AAD between 1 May 2011–30 November 2018 were studied. A total of 71 patients were identified with a history of ESRD, and 51 patients underwent dialysis therapy. AAD was discovered by computerized tomography in most patients (93%). The imaging modality of choice did not differ between the patients with and without ESRD for diagnosing aortic dissection. Baseline characteristics, comorbidities, laboratory and imaging findings, and clinical presentations of the patients with and without ESRD are shown in Tables 1 and 2. Male patients were dominant in both groups (71.8 and 77.4%). There were no significant differences in the distributions of age and gender. Hypertension was significantly more common among the patients with ESRD. However, smokers and alcoholic individuals were significantly less common among the patients with ESRD. In a laboratory test performed on admission, the patients with ESRD had lower leucocyte and platelet counts than the patients without ESRD, and this difference was statistically significant (*p* < 0.001). Typical presentations with chest or back pain, particularly those with an abrupt or migrating nature, occurred less commonly among the patients with ESRD. Type B aortic dissection was significantly more common among the patients with ESRD (76.1% versus 60.3%, *p* = 0.02).

**Table 1:** Demographics and Medical History of Patients With Acute Aortic Dissection

Variable	Overall	ESRD	No ESRD	*P*
N (%)	217 (100)	71 (32.7)	146 (67.3)	–
Demographics				
Age, mean (±SD)	56.7 (11.8)	57.7 (10.7)	54.6 (13.7)	0.07
Gender, Male(%)	164 (75.6)	51 (71.8)	113 (77.4)	0.37
Patient history				
Hypertension (%)	186 (85.7)	68 (95.8)	118 (80.8)	<0.01
Diabetes mellitus (%)	9 (4.1)	6 (8.5)	3 (2.1)	0.06
Cigarette (%)	106 (48.8)	21 (29.6)	85 (58.2)	<0.01
Alcoholic (%)	40 (18.4)	4 (5.6)	36 (24.7)	<0.01

*ESRD* End-stage renal disease

**Table 2:** Clinical Presentations, Signs, Diagnostic Imaging Examination, Laboratory findings on admission and Classification of Patients With Acute Aortic Dissection

Variable	Overall	ESRD	No ESRD	*P*
Clinical Presentations and Signs				
Typical pain (%)	165 (76.0)	43 (60.6)	122 (83.6)	<0.001
Atypical pain (%)	23 (10.6)	15 (21.1)	8 (5.5)	<0.001
Focal neurological deficits (%)	5 (2.3)	2 (2.8)	3 (2.1)	1.0
Other symptoms (%)	15 (6.9)	5 (7.0)	10 (6.8)	1.0
With no symptom (%)	9 (4.1)	6 (8.5)	3 (2.1)	0.064
Hypotension (%)	4 (1.8)	2 (2.8)	2 (1.4)	0.837
Mean systolic BP (SD), mmHg	156.9 (28.7)	161.9 (27.4)	154.4 (29.1)	0.069
Systolic BP over 180 mmHg(%)	51 (23.5)	23 (32.4)	28 (19.2)	0.031
Mean diastolic BP (SD), mmHg	86.7 (20.7)	89.2 (20.3)	85.5 (20.9)	0.223
Heart rate (SD), beats/min	81.3 (16.2)	81.7 (17.9)	81.1 (15.4)	0.796
Diagnostic Imaging				
Echocardiography (%)	5 (2.3)	1 (1.4)	4 (2.7)	0.896
Computerized tomography (%)	202 (93.1)	65 (91.5)	137 (93.8)	0.736
Magnetic resonance imaging (%)	8 (3.7)	5 (7.0)	3 (2.1)	0.148
Aortography (%)	2 (0.9)	0 (0)	2 (1.4)	1.0
Laboratory findings on admission				
SCr (SD), umol/L	346.9 (444.0)	887.7 (407.5)	83.9 (24.2)	<0.001
WBC (SD), 10^9 /L	10.4 (4.2)	7.9 (3.0)	11.6 (4.2)	<0.001
PLT (SD), 10^9 /L	199 (90.3)	170 (92.8)	213 (86.0)	<0.001
Classification of aortic dissection				
Stanford type A (%)	75 (34.6)	17 (23.9)	58 (39.7)	0.022
Stanford type B (%)	142 (65.4)	54 (76.1)	88 (60.3)	

*ESRD* End-stage renal disease; Typical pain: include chest pain, abdominal pain, and back pain, which is described as sharp or ripping pain particularly that with an abrupt or migrating nature; Atypical pain: physical pain except for typical pain

### Characteristics of renal disease in ESRD patients

The characteristics of renal disease, including treatment, in the ESRD group patients are shown in Table 3. The leading primary diseases causing ESRD were chronic glomerulonephritis, followed by hypertension, diabetes, ANCA-associated glomerulonephritis, IgA nephropathy, lupus nephritis, obstructive nephropathy, and polycystic kidney disease. The disease underlying ESRD was uncertain in 2 patients. Thirty-nine patients underwent hemodialysis, and 12 patients underwent peritoneal dialysis. The remaining 20 patients received medical therapy. In cases not on dialysis treatment, 15 patients refused RRT because they believed that their current quality of life, with their expected lifespan, outweighs the quality and quantity of life following RRT. The remaining 5 patients were recommended by their physician to follow a “watch-and-wait” approach (delayed start dialysis), since they had not yet developed uremic complications.

**Table 3:** Characteristics of the renal disease in the ESRD patients

Variable	ESRD Group
Primary cause of the end-stage renal disease	
Chronic glomerulonephritis(%)	36 (50.7)
Hypertension(%)	21 (29.6)
Diabetes(%)	4 (5.6)
ANCA-associated glomerulonephritis(%)	2 (2.8)
Lupus nephritis(%)	2 (2.8)
IgA nephropathy(%)	2 (2.8)
Polycystic kidney disease(%)	1 (1.4)
Obstructive nephropathy(%)	1 (1.4)
Unknown causes(%)	2 (2.8)
Type of dialysis	
Hemodialysis(%)	39 (54.9)
Peritoneal(%)	12 (16.9)

*ESRD* End-stage renal disease, *ANCA* Anti-neutrophil cytoplasmic antibodies

### In-hospital treatments and outcomes in AAD patients with and without ESRD

The patients’ management and hospital outcomes are shown in Table 4. The patients with ESRD were more likely to be managed medically compared with the patients without ESRD (71.8% versus 49.3%, *p* = 0.002). The length of hospitalization (survival to discharge) did not differ significantly between the two groups (17.1 versus 14.1; *p* = 0.079). The overall in-hospital mortality was 16.9% in the patients with ESRD and 10.3% in the patients without ESRD. There was no significant difference between the two groups for overall in-hospital mortality (*p* = 0.165), regardless of which therapeutic modality was chosen. Among the patients without ESRD, multivariate analysis revealed that type A aortic dissection (OR, 13.68; 95% CI, 1.92 to 98.90; *p* = 0.006) was an independent predictor of in-hospital mortality. However, no factor was identified as an independent predictor of in-hospital mortality in the patients with ESRD.

**Table 4:** In-Hospital Treatments and Outcomes of Patients With Acute Aortic Dissection

Variable	Overall	ESRD	No ESRD	*P*
Definitive Management				
Open or endovascular repair (%)	94 (43.3)	20 (28.2)	74 (50.7)	0.002
Medical management (%)	123 (56.7)	51 (71.8)	72 (49.3)	
Length of hospitalization (days)	15.0	17.1	14.1	0.079
Mortality (%)	27 (12.4)	12 (16.9)	15 (10.3)	0.165
Open or endovascular repair (%)	3 (3.2)	1 (5)	2 (2.7)	0.516
Medical management (%)	24 (19.5)	11 (21.6)	13 (18.1)	0.628

*ESRD* End-stage renal disease

## Discussion

From this study, we found that AAD patients with ESRD represent a unique population with important differences in their clinical manifestation, laboratory findings, and management. The three leading causes of ESRD were chronic glomerulonephritis, hypertension, and diabetes in this study.

For patients with ESRD, there are significant gender differences in the morbidity of AAD, with male patients having a higher incidence of AAD than female patients [7]. Our study demonstrated a similar finding for patients with ESRD. Hypertension and especially uncontrolled hypertension are the dominant risk factors for AAD [2]. In this study, more than 80 % of the patients had hypertension. Patients with ESRD have a higher prevalence of hypertension than those without ESRD. In addition, the ESRD group had a higher proportion of patients with systolic blood pressure over 180 mmHg on admission, which might be related to overactivation of the renin-angiotensin system and water-sodium retention in these patients [8]. Chest or back pain, which is the most common initial symptom of AAD, usually begins suddenly and is worst at the start [1]. However, our data indicated that patients with ESRD are less likely to present with abrupt onset chest or back pain. This is presumably a result of denervation of the cardiac sympathetic nervous system due to the significant comorbid conditions. Physicians should be aware of the atypical presentation of acute aortic tears in this cohort. Moreover, smokers and alcoholic individuals were significantly less common among the patients with ESRD in this study, which might be the result of aggressive self-control and follow-up.

Previous studies have demonstrated that inflammation plays an important role in AAD [9]. Platelet count was identified as an independent predictor of in-hospital mortality in a recent study [10]. Our data indicated that patients with ESRD have lower leucocyte and platelet counts than patients without ESRD on admission. However, leucocyte and platelet counts were not found to be independent predictors of in-hospital mortality by multivariate analysis.

For acute type A aortic dissection, urgent surgical repair is recommended to reduce the risk of life-threatening complications such as rupture, cardiac tamponade, severe aortic insufficiency, or stroke [11]. Indications for surgical intervention for patients with type B aortic dissection included rupture, aortic expansion, visceral malperfusion, and intractable pain [12]. In general, initial medical management has been the consensus for the treatment of acute type B aortic dissection unless associated with life-threatening complications. The optimal treatment of acute type B aortic dissection remains controversial [13, 14]. Our data indicated that patients with ESRD are less likely to be treated surgically and are more often managed by conservative medical strategies. Several potential explanations may be responsible for this less aggressive approach. On the one hand, type B aortic dissection was significantly more common among patients with ESRD in this study. On the other hand, patients with ESRD are known to have a shorter life expectancy than the average population and more comorbid conditions, thus leading to the refusal of surgery by the patient and/or family.

In general, AAD patients with ESRD have higher postprocedural morbidity and mortality rates compared with those without ESRD [15]. However, there was no significant difference between patients with and without ESRD for overall in-hospital mortality, regardless of which therapeutic modality was chosen. The underlying causes might be correlated with the higher proportion of type B aortic dissection in patients with ESRD, which offset the higher postprocedural mortality rate caused by ESRD.

## Conclusions

This study highlights differences in the clinical characteristics, management, and outcomes of AAD patients with ESRD. These patients usually have atypical symptoms and more comorbid conditions and are managed more conservatively. However, these patients have no in-hospital survival disadvantage over those without ESRD. Further studies are needed to better understand and optimize care for patients with ESRD presenting with AAD.

## Data Availability

Any Information related to this study is available from the corresponding author by email upon reasonable request.

## Abbreviations

AAD: Acute aortic dissection
ESRD: End-stage renal disease
